# The role of extracardiac radiotracer biodistribution in the evaluation of cardiac amyloidosis: a retrospective multicenter study on [99mTc]Tc-DPD and [99mTc]Tc-HMDP

**DOI:** 10.1007/s00259-026-08153-1

**Published:** 2026-10-23

**Authors:** Andrea Di Giorgio, Giacomo Battisti, Norma Bonazzi, Sonia Elisa Prisco, Maria Starace, Simone Longhi, Stefano Fanti, Valentina Berti, Francesco Cappelli, Rachele Bonfiglioli

**Affiliations:** 1 Nuclear medicine, Alma Mater Studiorum University of Bologna, Bologna, 40138 Italy; 2 Department of Nuclear Medicine, Azienda Unità Sanitaria Locale-IRCCS di Reggio Emilia, Reggio Emilia, Italy; 3 Nuclear Medicine Unit, Hospital Care Department of Radiology, Azienda Sanitaria Universitaria Giuliano Isontina, Trieste, Italy; 4 Nuclear Medicine Unit, Department of Experimental and Clinical Biomedical Sciences, University of Florence, Florence, Italy; 5 Cardiology Unit, Cardiac Thoracic and Vascular Department, IRCCS Azienda Ospedaliero-Universitaria di Bologna, Bologna, Italy; 6 Nuclear Medicine, IRCCS Azienda Ospedaliero-Universitaria di Bologna, Bologna, Italy; 7 Tuscan Regional Amyloidosis Centre, Careggi University Hospital, Florence, Italy

**Keywords:** Cardiac Amyloidosis, Bone Scintigraphy, Radiotracers Distribution, Perugini Score, Extracardiac Uptake

## Abstract

**Background:**

Bone scintigraphy using [99mTc]Tc-DPD or [99mTc]Tc-HMDP is a non-invasive cornerstone for diagnosing transthyretin cardiac amyloidosis (ATTR-CA). However, their interchangeability and the clinical relevance of systemic extracardiac uptake (ExtCU) remain poorly defined.

**Aim:**

To systematically compare the diagnostic performance and whole-body biodistribution profiles of [99mTc]Tc-DPD and [99mTc]Tc-HMDP in patients with suspected cardiac amyloidosis (CA).

**Methods:**

We retrospectively evaluated a multicenter cohort of patients investigated for suspected CA who underwent late planar whole-body scintigraphy and targeted SPECT/CT with either radiotracer as part of their diagnostic workup. Inclusion required a final, confirmed diagnosis of ATTRwt, ATTRv, or AL amyloidosis. Myocardial visual Perugini score (VISUAL_PS) and systemic ExtCU (pulmonary, appendicular muscular, visceral, and miscellaneous) were recorded.

**Results:**

The study included 224 patients (DPD: *n* = 109, comprising 70 ATTRwt, 20 ATTRv, 19 AL; HMDP: *n* = 115, comprising 90 ATTRwt, 13 ATTRv, 12 AL). In ATTRwt, the DPD cohort presented predominantly with Perugini Grade 2 (76%), whereas the HMDP cohort skewed toward Grade 3 (72%, *p* < 0.001). DPD was significantly associated with appendicular muscular uptake (ATTRwt: 84% vs. 38%, *p* < 0.001), creating a competitive “soft-tissue sink effect”. Conversely, HMDP demonstrated a unique predilection for pulmonary retention (ATTRwt: 68% vs. 46%, *p* = 0.008; ATTRv: 61% vs. 10%, *p* = 0.006). Visceral uptake was rare in ATTR but was a robust negative predictor strongly favoring AL amyloidosis (OR 0.016, *p* < 0.001).

**Conclusions:**

[99mTc]Tc-DPD and [99mTc]Tc-HMDP possess distinct biodistribution profiles precluding direct interchangeability. HMDP maximizes myocardial contrast, whereas DPD provides a superior representation of the systemic musculoskeletal amyloid burden. Nuclear medicine physicians must adopt tracer-specific interpretive approaches to optimize non-invasive phenotyping.

**Trial registration:**

not applicable.

## Introduction and aims

Historically, systemic amyloidosis with cardiac involvement was classified as a rare and inexorably fatal condition, often diagnosed at advanced stages [1]. However, the advent of straightforward non-invasive diagnostic algorithms and significantly increased clinical awareness have radically shifted this paradigm. Current epidemiological data indicate that the prevalence of the two most common etiologies, transthyretin amyloidosis (ATTR), occurring in either wild-type (ATTRwt) or variant (ATTRv) forms, and acquired monoclonal immunoglobulin light-chain amyloidosis (AL), is substantially higher than previously recognized, transforming cardiac amyloidosis from an orphan disease to a prominent clinical challenge in modern cardiology [2, 3].

This epidemiological shift has coincided with a therapeutic revolution [4]. The development and approval of disease-modifying therapies, capable of stabilizing the amyloidogenic precursor and slowing disease progression, have dramatically altered the natural history and improved the survival of ATTR patients. Furthermore, recent years have witnessed the introduction of novel therapeutic classes, including RNA-silencing agents and antisense oligonucleotides (ASOs), which have further enhanced ATTR patient outcomes. Similarly, the prognosis of AL amyloidosis has been radically transformed by the introduction of novel plasma cell–directed therapies, such as anti-CD38 monoclonal antibodies and proteasome inhibitors, which effectively target the underlying amyloid-producing clone [5–7]. Consequently, there is an emerging and critical need to identify reliable clinical indicators capable of staging the disease, guiding therapeutic decision-making, and personalizing treatment strategies [8]. In this context, accurately assessing the extent of the total amyloid burden has become crucial [9]. Recent literature underscores this necessity, highlighting that precise metrics of myocardial infiltration, such as myocardial extracellular volume (ECV) quantified via Cardiac Magnetic Resonance Imaging (cMRI), serve as powerful and independent mortality predictors in both ATTR and AL amyloidosis [10].

In the current diagnostic landscape, bone scintigraphy using technetium-labeled tracers [99mTc]Tc-PYP, [99mTc]Tc-HMDP, and [99mTc]Tc-DPD plays a pivotal, non-invasive role [11]. While the exact mechanisms underlying the in vivo localization of these bone-seeking diphosphonates in amyloid deposits are not yet fully elucidated, it is widely postulated that tracer retention is calcium-mediated. Current hypotheses suggest that diphosphonates bind to microcalcifications and higher calcium concentrations specifically harbored within the structure of transthyretin amyloid fibrils, which explains their marked affinity for ATTR compared to AL cardiomyopathy. The primary diagnostic power of these osteotropic agents lies in their ability to demonstrate intense cardiac uptake, which, in the absence of a monoclonal gammopathy, is highly specific for confirming ATTR cardiomyopathy without the need for endomyocardial biopsy [12]. However, because amyloidosis is inherently a systemic disease, the utility of these radiotracers extends beyond the myocardium. During routine imaging, noticeable extracardiac radiotracer accumulation is frequently observed in soft tissues, including the lungs, liver, spleen, and skeletal muscle [13].

Despite the high diagnostic accuracy of bone scintigraphy, the exact clinical and pathophysiological significance of this non-myocardial uptake remains poorly defined. While the cardiac signal is rigorously quantified using grading systems such as the Perugini score (VISUAL_PS) (Table 1), the extracardiac biodistribution is often relegated to an incidental finding [14]. Furthermore, different radiotracers are currently utilized interchangeably across clinical centers, despite emerging evidence suggesting that their specific pharmacokinetics and affinities for amyloid deposits may yield distinct whole-body imaging phenotypes [9, 15]. Understanding these nuanced biodistribution profiles is essential, as the diffusion of radiotracers into non-myocardial tissues may hold untapped potential for differential diagnosis and systemic disease staging.

**Table 1:** The Perugini visual grading system and diagnostic workflow for cardiac amyloidosis

Clinical Suspicion of Cardiac Amyloidosis	Perugini Grade	Scintigraphic Findings (Cardiac vs. Bone Uptake)	Clinical Interpretation
	All Grades (0–3) *(Preliminary Step)*	Applicable to all grades	Mandatory Rule-out of AL Amyloidosis: Before interpreting any grade, exclude AL cardiac amyloidosis by performing all 3 tests: serum and urine immunofixation with protein electrophoresis, and serum free light chain assay.
	Grade 0	No cardiac uptake and normal bone uptake.	ATTR cardiac amyloidosis unlikely (consider genetic testing to exclude ATTRv forms that may be negative on bone scintigraphy, e.g., Val30Met).
	Grade 1	Mild cardiac uptake, distinctly lower in intensity than normal bone uptake.	Early-phase ATTR cardiac amyloidosis is possible, but further investigation is mandatory (histopathological confirmation).
	Grade 2–3 ($\:\ge\:2$)	Moderate-to-high cardiac uptake equal to or greater than bone uptake.	ATTR cardiac amyloidosis diagnosis can be established without histopathological examination. Genetic testing is required regardless of age.

Therefore, the aim of this multicenter retrospective study is to systematically evaluate the diagnostic performance and specific whole-body biodistribution profiles of two distinct radiotracers, [99mTc]Tc-DPD and [99mTc]Tc-HMDP, in a real-world clinical population with a confirmed final diagnosis encompassing various forms of systemic amyloidosis. By analyzing both the intensity of myocardial positivity and the distinct patterns of extracardiac radiotracer distribution, this study seeks to elucidate whether non-myocardial tissue retention carries specific clinical and pathological significance, ultimately refining the interpretive approach to these critical imaging modalities.

## Materials and methods

Multicenter retrospective analysis involving patients with a confirmed diagnosis of CA. The study cohort was independently enrolled from two Italian referral centers, Bologna and Florence, each utilizing a specific and different radiotracer for diagnostic evaluation. The choice of radiotracer between centers reflected institutional research backgrounds and regulatory frameworks in Italy: the Bologna cohort utilized [99mTc]Tc-DPD, aligning with extensive ongoing institutional research protocols involving this agent, whereas the Florence cohort utilized [99mTc]Tc-HMDP, representing standard clinical practice in compliance with national regulatory labeling, as it is currently the only bone-seeking radiotracer holding a formal indication for amyloidosis in its official Italian datasheet. In particular, the Bologna Cohort included patients evaluated with 99mTc-3,3-diphosphono-1,2-propanodicarboxylic acid (DPD) scintigraphy, while the Florence Cohort comprised patients evaluated with 99mTc-hydroxymethylene diphosphonate (HMDP) scintigraphy. For all participants, demographic data, clinical characteristics, and imaging findings were systematically collected. The definitive diagnosis of CA was established based on consensus clinical, laboratory, histopathological, and imaging criteria, leading to classification into three primary groups: ATTRwt, ATTRv, and AL amyloidosis [16]. The study protocol was approved by the Institutional Ethics Committees of both centers and was conducted in accordance with the ethical principles of the Declaration of Helsinki.

### Scintigraphy protocol and image acquisition

All patients underwent late planar Whole-Body (WB) scintigraphy, acquired approximately 180 min following the intravenous administration of 740 MBq of the respective radiopharmaceutical (DPD or HMDP), in adherence to current procedural guidelines [17]. Fasting was not required prior to the examination; however, patients were instructed to maintain adequate hydration post-injection and to void their bladder immediately before scanning to minimize pelvic background activity.

Imaging was performed using a dual-head SPECT/CT gamma camera (Discovery NM/CT 670, GE Healthcare in Bologna Cohort, and Discovery NM/CT 870, GE Healthcare in Florence cohort) equipped with Low-Energy High-Resolution (LEHR) collimators. Planar whole-body images were acquired in anterior and posterior views using a 256 × 256 matrix with a continuous scanning speed of 10 to 15 cm/min.

Dedicated SPECT/CT acquisitions were performed where indicated to confirm the precise myocardial location of uptake and anatomically characterize extracardiac findings according to EANM guidelines. SPECT acquisitions were performed in a step-and-shoot mode with automatic body contouring, utilizing a 180° detector configuration per head. Emission data were collected in a 128 × 128 matrix with 60 projections (20 s per projection). Images were reconstructed using an iterative Ordered-Subset Expectation Maximization (OSEM) algorithm. A low-dose, non-diagnostic CT scan was performed sequentially for attenuation correction and anatomical localization. CT parameters included a tube voltage of 120 kVp and a tube current of 30–50 mA with automatic dose modulation.

## Nuclear medicine image evaluation

Image interpretation was conducted locally by a team of three experienced nuclear medicine physicians at each center, defined as specialists with at least 8 to 10 years of specific clinical experience in reporting whole-body bone scintigraphy and targeted SPECT/CT studies for CA. Prior to study initiation, a joint calibration and training session was conducted using a pilot set of cases to harmonize VISUAL_PS, Janssen Score (JS) and extracardiac evaluation. All readers were blinded to the final clinical diagnosis and patient identity. Myocardial radiotracer uptake was qualitatively assessed on planar images using VISUAL_PS [14]. This grading system ranks cardiac retention in relation to bone uptake, where grade 0 indicates no cardiac activity and grade 1 represents mild uptake that remains less intense than the skeletal signal. Grade 2 is defined by moderate cardiac uptake equal to the bone signal, often accompanied by a perceived reduction in skeletal intensity, while grade 3 reflects intense myocardial retention with minimal or absent bone uptake [14].

Beyond the standard myocardial assessment, the presence and intensity of tracer retention in sites outside the heart and physiological skeleton, collectively defined as Extracardiac Uptake (ExtCU), were systematically evaluated primarily on planar whole-body images, utilizing SPECT/CT imaging to strictly confirm anatomical localization when necessary. Specific sites of interest included pulmonary involvement, quantified using the established JS, uptake in the appendicular musculature (MU), and activity in the abdominal viscera, specifically the liver and spleen [18]. Any additional relevant findings, such as uptake in the tongue or salivary glands, were categorized as Miscellaneous Uptake (MiscU). The overall presence of ExtCU in any anatomical site was recorded as a binary variable. To ensure data robustness, a subset of random scans from each cohort underwent interobserver variability analysis via blinded re-evaluation by nuclear medicine physicians from the collaborating center.

### Statistical analysis

Statistical analyses were conducted using R software (version 4.3.1) and Python libraries, with statistical significance defined by a two-sided p-value of less than 0.05. Continuous demographic variables, such as age, were expressed as median and interquartile range (IQR). Given the non-normal distribution and heterogeneity of the subgroups, the distribution of age across diagnostic groups and between the two centers (DPD vs. HMDP) was compared using the non-parametric Mann-Whitney U test and the Kruskal-Wallis H test, respectively. Comparisons of categorical variables, including gender, VISUAl_PS, JS, and binary ExtCU parameters, were performed using the Chi-Square test of independence for contingency tables larger than 2 × 2, and Fisher’s exact test for 2 × 2 comparisons. To determine the predictive value of scintigraphic findings, multivariate logistic regression models were constructed separately for the DPD and HMDP cohorts, thereby isolating the independent effect of each radiotracer. Three primary binary outcomes were modeled to differentiate between groups: ATTRwt versus AL, ATTRv versus AL, and ATTRwt versus ATTRv. These models incorporated structural imaging parameters as independent predictors, specifically a high VISUAL_PS (defined as ≥ 2), the presence of MU, MiscU ≥ 1, and visceral uptake. Results were reported as Odds Ratios (OR) with 95% Confidence Intervals (CI).

## Results

### Patient characteristics and demographic statistics

The study included a total of 224 patients with a confirmed diagnosis of cardiac amyloidosis and complete diagnostic data, recruited consecutively between 2015 and 2021. The study population was stratified into two cohorts based on the radiotracer used: the Bologna cohort, evaluated with [99mTc]Tc-DPD (*n* = 109), and the Florence cohort, evaluated with [99mTc]Tc-HMDP (*n* = 115) (Table 2). The overall population was predominantly composed of patients with Wild-Type Transthyretin Amyloidosis (ATTRwt), which accounted for 71% (*n* = 160) of cases, followed by Hereditary Transthyretin Amyloidosis (ATTRv) (15%, *n* = 33) and AL Amyloidosis (14%, *n* = 31).

**Table 2:** Baseline demographic, clinical, and scintigraphic characteristics of the study population stratified by radiotracer

Characteristics	All patients	DPD cohort	HMDP cohort	*p*-value
*n.*	*224*	*109*	*115*	
Age (y)	79 (72–83.4)	77 (68–82)	80 (73.5–84)	0.005
Sex				
Male	186 (83%)	91 (83,5%)	95 (83%)	1
Female	38 (17%)	18 (16.5%)	29 (17%)	1
ATTRwt	160 (71%)	70 (64%)	90 (78%)	0.003
ATTRv	33 (15%)	20 (18%)	13 (11%)	0.194
AL	31 (14%)	19 (18%)	12 (11%)	0.186
VISUAL_PS				0.001
0	22 (10%)	13 (12%)	9 (8%)	
1	14 (6%)	6 (6%)	8 (7%)	
2	93 (42%)	68 (62%)	25 (22%)	
3	95 (42%)	22 (20%)	73 (63%)	
ExtCu	202 (90%)	90 (83%)	100 (87%)	0.461
Positive Pulmonary Uptake	109 (49%)	37 (34%)	72 (63%)	< 0.001
JS				< 0.001
0	115 (51%)	72 (66%)	43 (37%)	
1	70 (31%)	15 (14%)	55 (48%)	
2	39 (18%)	22 (20%)	17 (15%)	
Positive MU	122 (54%)	79 (73%)	43 (37%)	< 0.001
MiscU				< 0.001
0	62 (28%)	27 (24%)	35 (30%)	
1	75 (33%)	50 (46%)	25 (22%)	
2	2 (1%)	2 (2%)	0 (0%)	
3	26 (12%)	26 (24%)	0 (0%)	
4	13 (6%)	2 (2%)	11 (10%)	
5	46 (20%)	2 (2%)	44 (38%)	

*Data are expressed as n (%) or median (interquartile range). ATTRwt: wild-type transthyretin amyloidosis; ATTRv: hereditary transthyretin amyloidosis; AL: light-chain amyloidosis; VISUAL_PS: Perugini visual score; ExtCu: overall extracardiac uptake; JS: Janssen score; MU: muscular uptake; MiscU: miscellaneous uptake.*

The distribution of these diagnostic groups differed significantly between the two centers (*p* = 0.017). Specifically, the Florence cohort contained a higher proportion of ATTRwt patients (78%, *n* = 90) compared to the Bologna cohort (64%, *n* = 70). The prevalence of ATTRv was 18% (*n* = 20) in the Bologna cohort and 11% (*n* = 13) in the Florence cohort.

Regarding sex distribution, the cohorts were homogeneous (*p* = 1.000), with a marked male predominance observed in both the DPD group (83.5% males) and the HMDP group (83% males).

The median age was comparable between centers for patients with ATTRwt (*p* = 0.175) and AL amyloidosis (*p* = 0.478). The statistically significant difference was observed in the overall population (*p* = 0.005) and specifically within the ATTRv subgroup (*p* = 0.004). Patients with ATTRv in the Bologna cohort were significantly younger (median 64.2 years, IQR 55.4–76.5) compared to those in the Florence cohort (median 79.0 years, IQR 72.0–82.0).

### Scintigraphic comparison and tracer biodistribution

Comparative analysis demonstrated statistically significant differences in the biodistribution of [99mTc]Tc-DPD and [99mTc]Tc-HMDP across ATTR subtypes (Tables 3 and 4).

**Table 3:** Demographic and scintigraphic characteristics of the ATTRwt subgroup stratified by radiotracer

Characteristics	All patients	DPD cohort	HMDP cohort	*p*-value
n.	*160*	*70*	*90*	
Age (y)	81 (76.6–84.8)	78.6(72.2–83.7)	81(77.3–85)	0.175
Sex				
Male	143 (89%)	64 (91%)	79 (88%)	0.607
Female	17 (11%)	6 (9%)	11 (12%)	0.607
VISUAL_PS				< 0.001
0	0 (0%)	0 (0%)	0 (0%)	
1	10 (6%)	2 (3%)	8 (9%)	
2	70 (44%)	53 (76%)	17 (19%)	
3	80 (50%)	15 (21%)	65 (72%)	
ExtCu	146 (91%)	66 (94%)	80 (89%)	0.359
Positive Pulmonary Uptake	93 (58%)	32 (46%)	61 (68%)	0.008
JS				< 0.001
0	67 (42%)	38 (54%)	29 (32%)	
1	60 (37%)	13 (19%)	47 (52%)	
2	33 (21%)	19 (27%)	14 (16%)	
Positive MU	93 (58%)	59 (84%)	34 (38%)	< 0.001
MiscU				< 0.001
0	31 (19%)	8 (11%)	23 (26%)	
1	63 (39%)	41 (59%)	22 (24%)	
2	2 (1%)	2 (3%)	0 (0%)	
3	15 (10%)	15 (22%)	0 (0%)	
4	12 (8%)	2 (3%)	10 (11%)	
5	37 (23%)	2 (3%)	35 (39%)	

*Data are expressed as n (%) or median (interquartile range). ATTRwt: wild-type transthyretin amyloidosis; VISUAL_PS: Perugini visual score; ExtCu: overall extracardiac uptake; JS: Janssen score; MU: muscular uptake; MiscU: miscellaneous uptake.*

**Table 4:** Demographic and scintigraphic characteristics of the ATTRv subgroup stratified by radiotracer

Characteristics	All patients	DPD cohort	HMDP cohort	*p*-value
n.	*33*	*20*	*13*	
Age (y)	76(63.4–79)	64.2(52.3–76.8)	79(77–81)	0.004
Sex				
Male	20 (61%)	13 (65%)	7 (35%)	0.717
Female	13 (39%)	7 (54%)	6 (46%)	0.717
VISUAL_PS				0.103
0	3 (9%)	3 (15%)	0 (0%)	
1	0 (0%)	0 (0%)	0 (0%)	
2	20 (61%)	13 (65%)	7 (54%)	
3	10 (30%)	4 (20%)	6 (46%)	
ExtCu	29 (88%)	17 (85%)	12 (92%)	0.934
Positive Pulmonary Uptake	10 (30%)	2 (10%)	8 (61%)	0.006
JS				0.003
0	23 (70%)	18 (90%)	5 (38.5%)	
1	5 (15%)	0 (0%)	5 (38.5%)	
2	5 (15%)	2 (10%)	2 (10%)	
Positive MU	21 (64%)	16 (80%)	5 (38.5%)	0.04
MiscU				< 0.001
0	7 (21%)	4 (20%)	3 (23%)	
1	10 (31%)	8 (40%)	2 (15%)	
2	0 (0%)	0 (0%)	0 (0%)	
3	8 (24%)	8 (40%)	0 (0%)	
4	1 (3%)	0 (0%)	1 (8%)	
5	7 (21%)	0 (0%)	7 (54%)	

*Data are expressed as n (%) or median (interquartile range). ATTRv: hereditary transthyretin amyloidosis; VISUAL_PS: Perugini visual score; ExtCu: overall extracardiac uptake; JS: Janssen score; MU: muscular uptake; MiscU: miscellaneous uptake.*

**Fig. 1:**
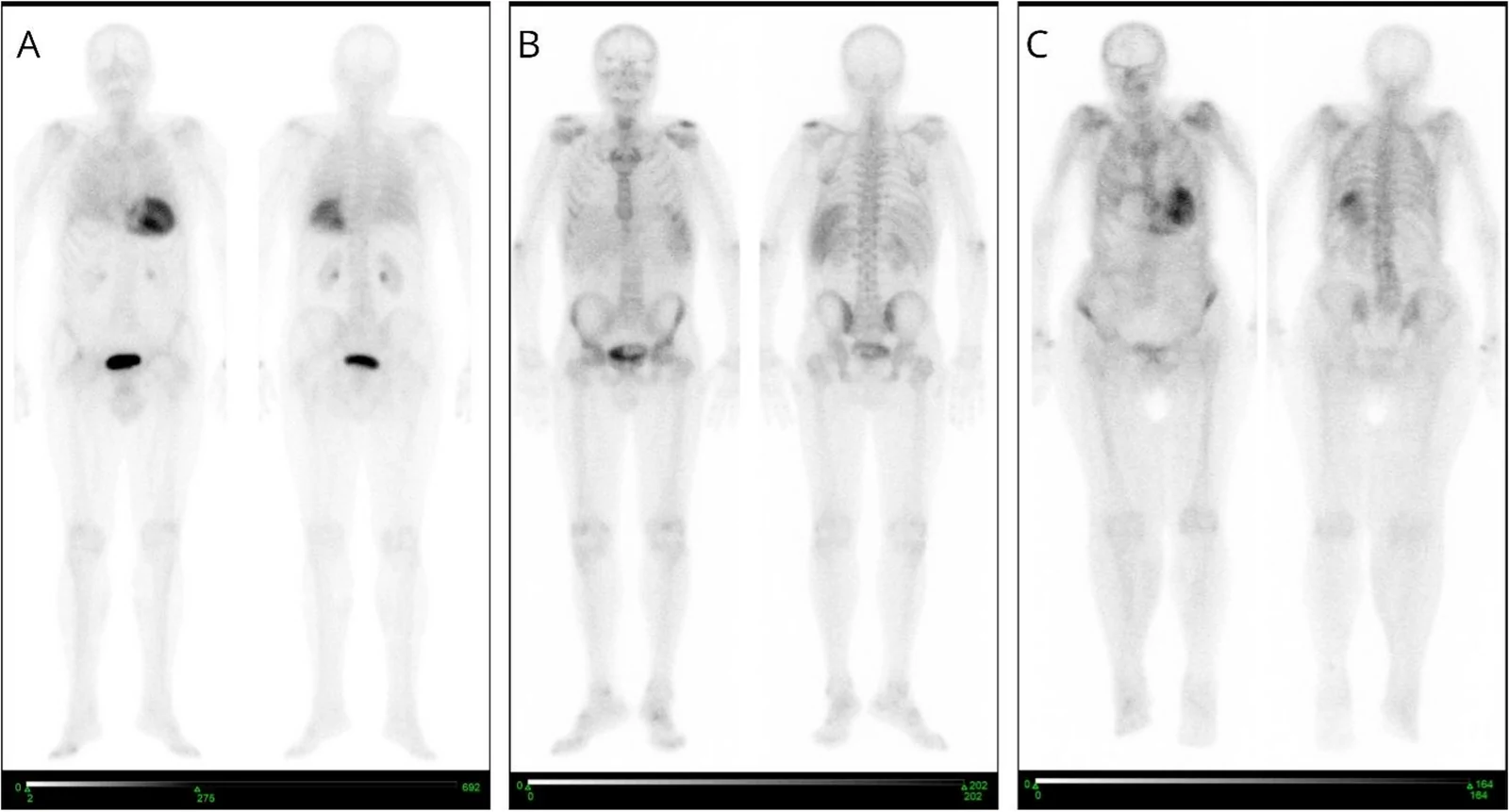
^[99mTc]Tc−DPD^ extracardiac biodistribution phenotypes in the Bologna cohort. (**A**) ATTRv subtype: Whole-body scan showing intense biventricular cardiac uptake accompanied by atypical pulmonary tracer retention (Janssen Score ≥ 1) and diffuse soft-tissue fixation across the appendicular, thoracic, and abdominal regions. (**B**) AL amyloidosis: hepato-splenic visceral uptake acting as a tracer-independent “red flag” for light-chain disease, with minimal myocardial avidity. (**C**) ATTRwt subtype: Miscellaneous scans demonstrating biventricular cardiac uptake with diffuse soft-tissue fixation limited to the appendicular, thoracic, and abdominal regions

**Fig. 2:**
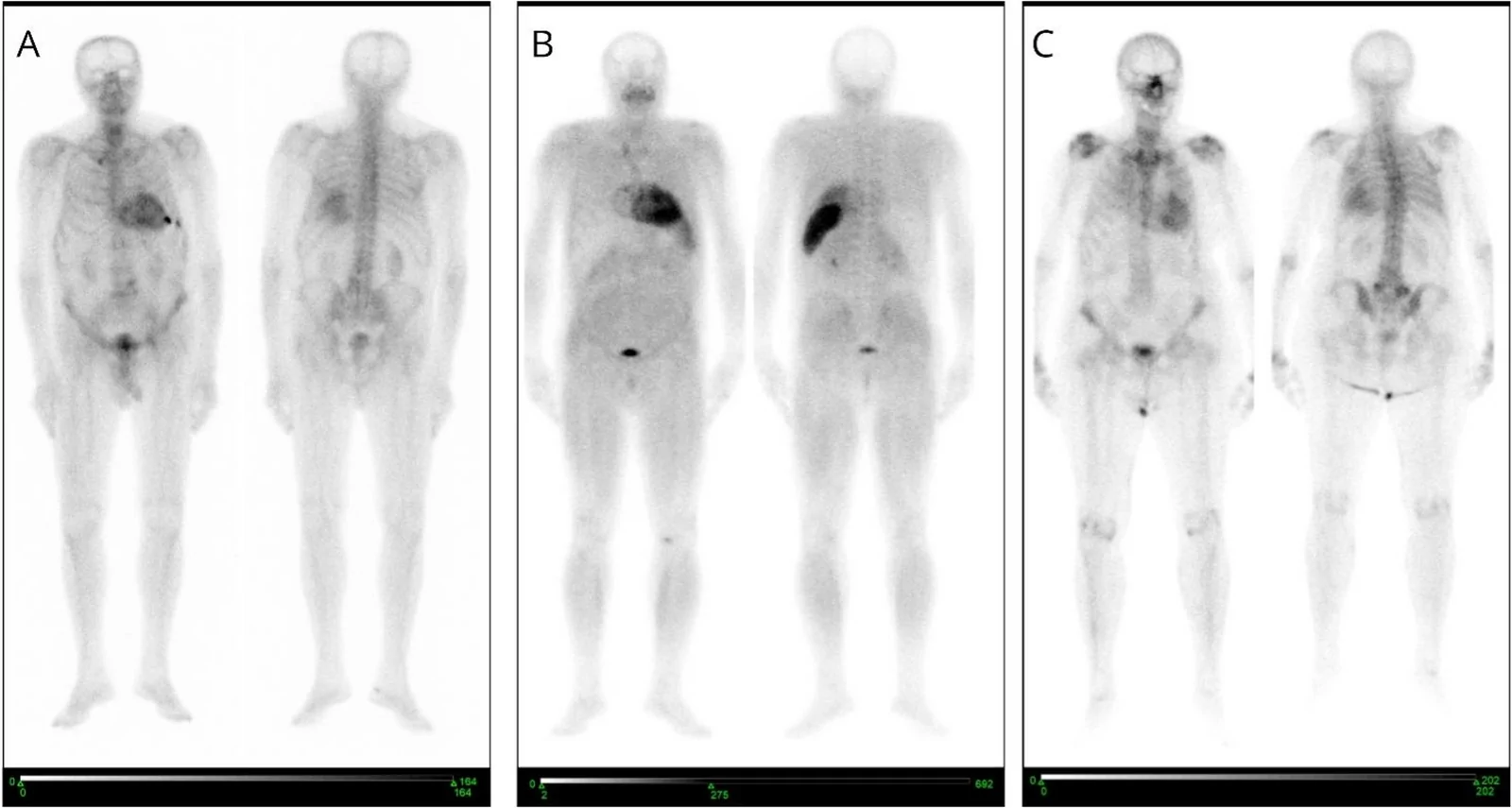
[99mTc]Tc-HMDP whole-body scintigraphic phenotypes observed in the Florence cohort. (**A**) ATTRv subtype: Representative scan showing intense biventricular cardiac uptake accompanied by pulmonary tracer retention (Janssen Score ≥ 1) and soft-tissue fixation in the appendicular and abdominal regions. An incidental focal hotspot corresponding to a traumatic rib fracture is noted in the left rib cage. (**B**) AL amyloidosis: An uncommon case of light-chain disease presenting with intense biventricular cardiac retention, marked pulmonary tracer accumulation, and soft-tissue fixation across the appendicular and abdominal zones. (**C**) ATTRwt subtype: Scan showing the typical [99mTc]Tc-HMDP biodistribution profile, characterized by intense cardiac uptake, prominent pulmonary retention (Janssen Score$\:\ge\:$1), and soft-tissue fixation restricted to the appendicular and abdominal regions

In the ATTRwt subgroup, the DPD cohort showed a predominant prevalence of Score 2 (76%, *n* = 53) compared to Score 3 (21%, *n* = 15). Conversely, the HMDP cohort was characterized by a significantly higher prevalence of Score 3 (72%, *n* = 65) compared to Score 2 (19%, *n* = 17). The VISUAL_PS distribution was not significantly different in the AL group (*p* = 0.367), where the majority of patients presented with Score 0 or 1 regardless of the radiotracer used (Table 5) (Figs. 1 and 2).

**Table 5:** Demographic and scintigraphic characteristics of the AL subgroup stratified by radiotracer

Characteristics	All patients	DPD cohort	HMDP cohort	*p*-value
n.	*31*	*19*	*12*	
Age (y)	69 (60-74.8)	73.9 (58.8–76.3)	68.5(60–72)	0.478
Sex				
Male	23 (74%)	14 (74%)	9 (75%)	1
Female	8 (26%)	5 (26%)	3 (25%)	1
VISUAL_PS				0.367
0	19 (61%)	10 (53%)	9 (75%)	
1	4 (13%)	4 (21%)	0 (0%)	
2	3 (10%)	2 (10%)	1 (8%)	
3	5 (16%)	3 (16%)	2 (17%)	
ExtCu	15 (48%)	7 (37%)	8 (67%)	0.211
Positive Pulmonary Uptake	6 (19%)	3 (16%)	3 (25%)	0.868
JS				0.435
0	25 (81%)	16 (84%)	9 (75%)	
1	5 (16%)	2 (11%)	3 (25%)	
2	1 (3%)	1 (5%)	0 (0%)	
Positive MU	8 (26%)	4 (21%)	4 (33%)	0.734
MiscU				0.159
0	24 (78%)	15 (79%)	9 (75%)	
1	2 (6,5%)	1 (5%)	1 (8%)	
2	0 (0%)	0 (0%)	0 (0%)	
3	3 (10%)	3 (16%)	0 (0%)	
4	0 (0%)	0 (0%)	0 (0%)	
5	2 (6.5%)	0 (0%)	2 (17%)	

*Data are expressed as n (%) or median (interquartile range). AL: light-chain amyloidosis; VISUAL_PS: Perugini visual score; ExtCu: overall extracardiac uptake; JS: Janssen score; MU: muscular uptake; MiscU: miscellaneous uptake.*

In patients with ATTRwt, pulmonary uptake was observed in 68% (*n* = 61) of the HMDP cohort versus 46% (*n* = 32) of the DPD cohort (*p* = 0.008). In the ATTRv group, pulmonary uptake was detected in 61% (*n* = 8) of patients evaluated with HMDP versus 10% (*n* = 2) of those evaluated with DPD (*p* = 0.006) (Fig. 2).

In the ATTRwt subgroup, uptake in the appendicular musculature was observed in 84% (*n* = 59) of DPD patients compared to 38% (*n* = 34) of HMDP patients (*p* < 0.001). A similar trend was observed in the ATTRv subgroup (*p* = 0.040), where MU was present in 80% (*n* = 16) of DPD scans versus 38.5% (*n* = 5) of HMDP scans.

MiscU was significantly more frequent in the DPD cohort for ATTRwt patients (89% vs. 74%, *p* = 0.027) but showed no statistically significant difference in the ATTRv subgroup (*p* = 0.287).

**Fig. 3:**
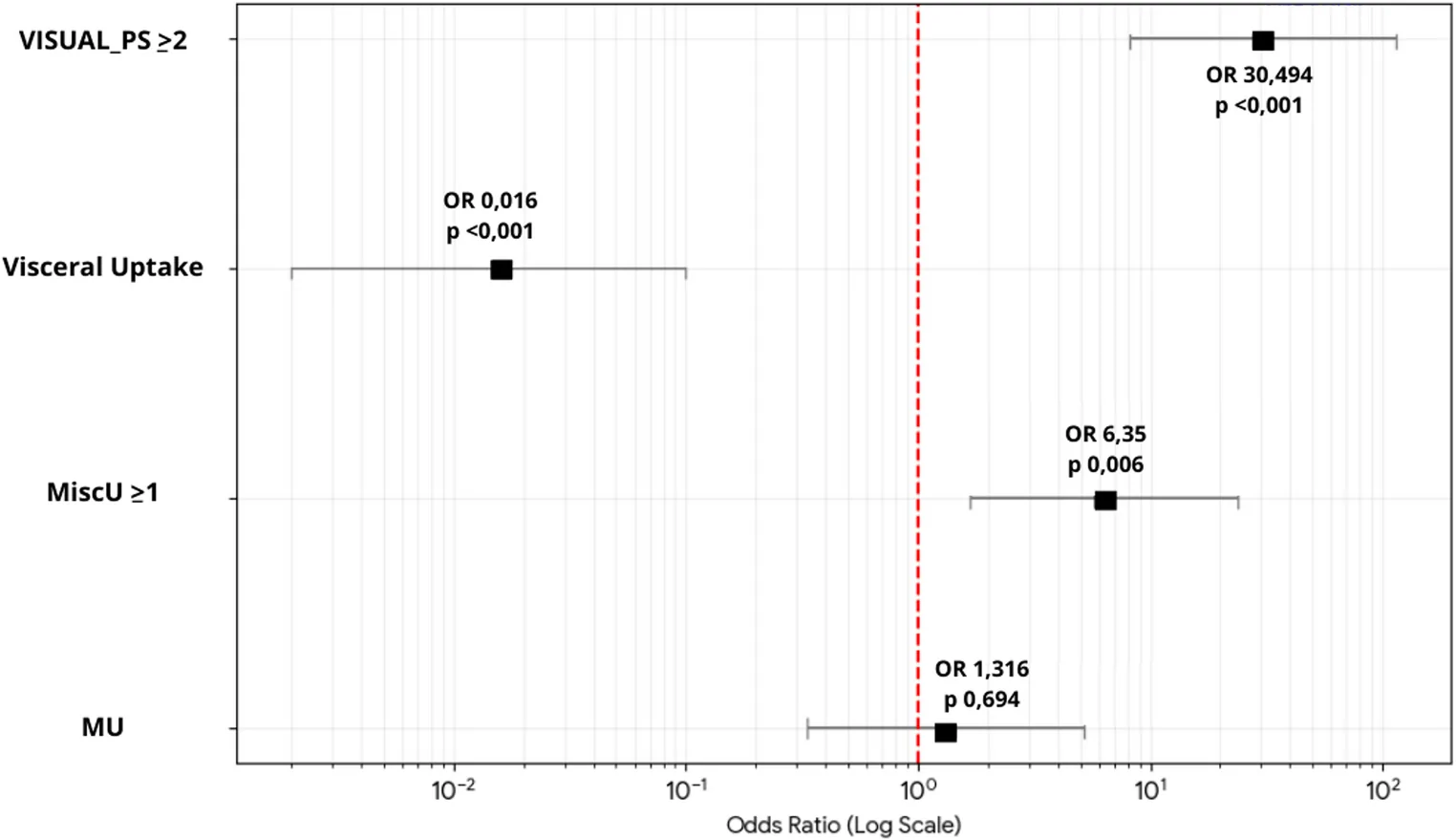
Forest plot summarizing the multivariate logistic regression analysis for the prediction of ATTR amyloidosis. The diagram illustrates the Odds Ratios (OR) and 95% Confidence Intervals (95% CI) for key scintigraphic findings, where solid squares represent the point estimate of the OR and horizontal bars indicate the 95% CI relative to the vertical dashed line of no effect at OR = 1. The analysis demonstrates that a VISUAL_PS (≥ 2) and MiscU (≥ 1) are significant independent predictors associated with an increased likelihood of ATTR diagnosis, as indicated by their position to the right of the reference line. Conversely, Visceral Uptake is strongly associated with a reduced likelihood of ATTR, thereby favoring an AL diagnosis, while MU is shown to be a non-significant discriminator in this model as its confidence interval intersects the vertical reference line. Note that the x-axis is presented on a logarithmic scale to accommodate the wide range of odds ratios

Visceral Uptake (involving liver or spleen) was a rare finding in ATTR patients across both cohorts, with no statistically significant difference in prevalence between the DPD and HMDP groups (*p* = 0.812). However (Figs. 1A and 2B), visceral uptake was significantly more common in the AL group compared to both ATTR subgroups (*p* < 0.001), regardless of the tracer used, confirming its role as a robust negative predictor for ATTR diagnosis (OR 0.016, Fig. 3).

## Scintigraphic Predictors of ATTR Diagnosis

Multivariate logistic regression models were performed to identify independent scintigraphic predictors of ATTR diagnosis (vs. AL) and to evaluate differences between tracers.

In the overall population, a VISUAL_PS ≥ 2 was identified as a significant independent predictor of ATTR diagnosis (OR 30.49, 95% CI 8.16–113.93, *p* < 0.001). Similarly, MiscU was significantly associated with ATTR diagnosis (OR 6.35, 95% CI 1.68–23.97, *p* = 0.006). Conversely, Visceral Uptake was strongly associated with a lower likelihood of ATTR, favoring a diagnosis of AL amyloidosis (OR 0.016, 95% CI 0.002–0.100, *p* < 0.001). MU was not a statistically significant predictor in the multivariate model (OR 1.32, *p* = 0.694) (Fig. 3).

**Fig. 4:**
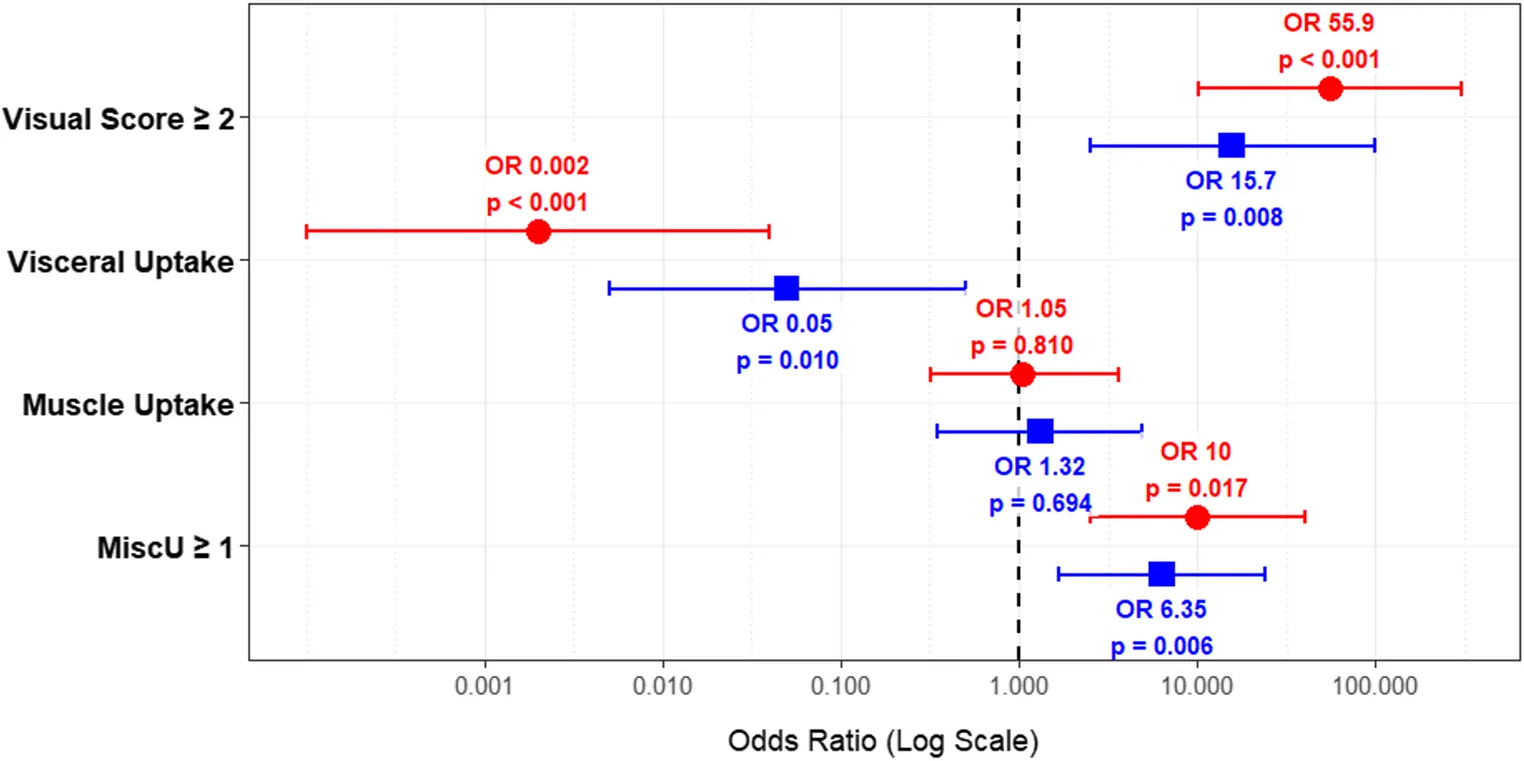
Comparative forest plot of the multivariate logistic regression analysis for the prediction of ATTR amyloidosis (wild-type and variant) versus AL amyloidosis. The chart displays the Odds Ratios (OR) and corresponding 95% confidence intervals for the [99mTc]Tc-DPD cohort (blue squares) and the [99mTc]Tc-HMDP cohort (red circles). The analysis demonstrates that a VISUAL_PS ≥ 2 and MiscU ≥ 1 are significant independent predictors associated with an increased likelihood of ATTR diagnosis. Notably, while VISUAL_PS ≥ 2 is a strong predictor in both groups, it exhibits a more pronounced association in the HMDP cohort. Conversely, Visceral Uptake is robustly associated with an AL diagnosis (acting as a negative predictor for ATTR) across both tracers. Muscle Uptake (MU) did not reach statistical significance as an independent predictor in these models, as its confidence intervals intersect the vertical reference line of no effect (OR = 1). Note that the x-axis is presented on a logarithmic scale

When stratified by radiotracer, multivariate logistic regression model showed that VISUAL_PS ≥ 2 was a significant predictor in both cohorts, albeit with higher OR in the HMDP model (OR 55.85, *p* < 0.001) compared to the DPD model (OR 15.72, *p* = 0.008). Conversely, Visceral Uptake remained a significant negative predictor for ATTR in both the DPD cohort (OR 0.05, *p* = 0.010) and the HMDP cohort (OR 0.002, *p* < 0.001) (Fig. 4).

**Fig. 5:**
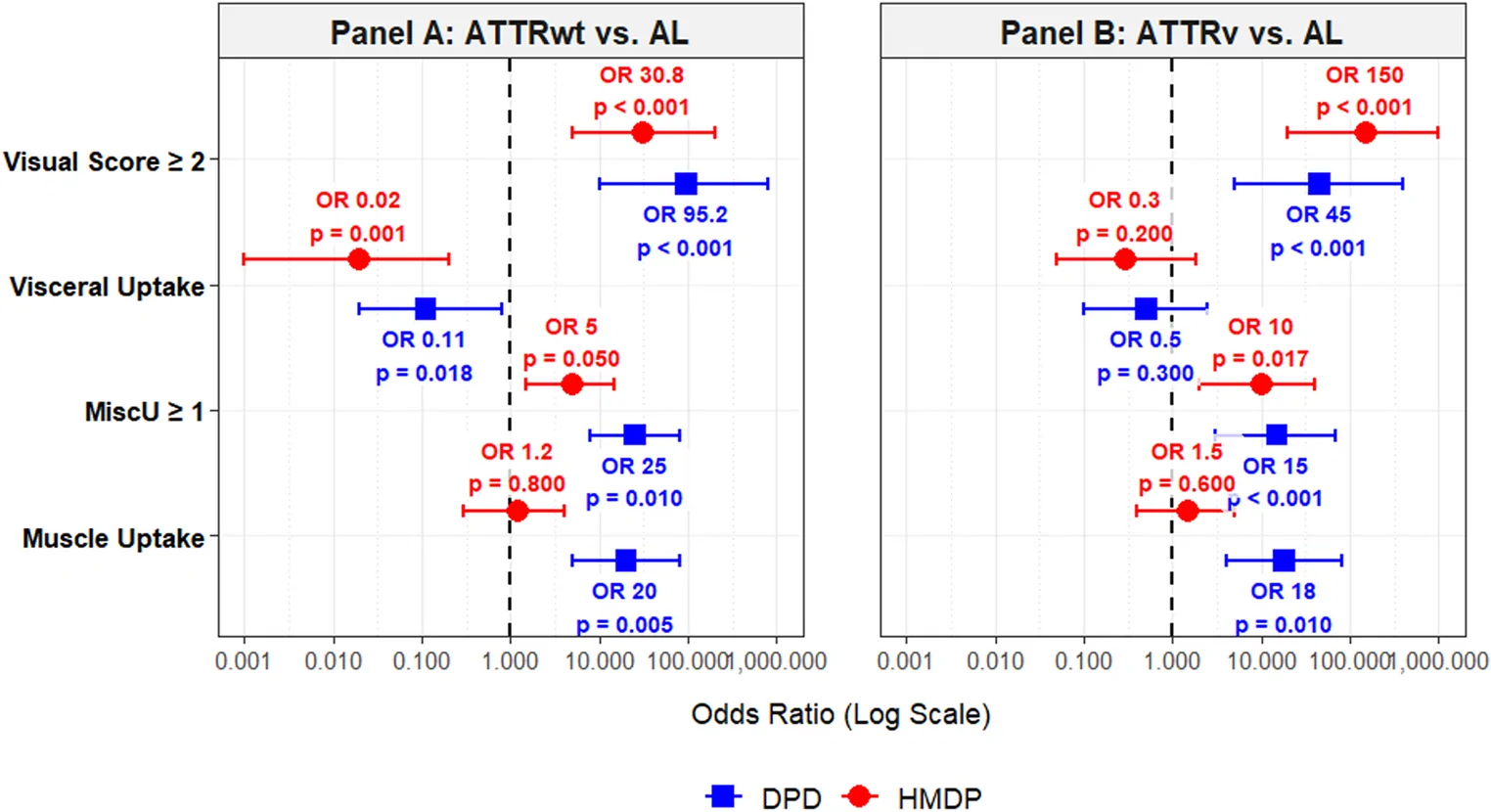
Subgroup analysis of scintigraphic predictors for ATTRwt (Panel **A**) and ATTRv (Panel **B**) diagnoses. In Panel A, both tracers show comparable and robust predictive patterns for the ATTRwt phenotype, with High VISUAL_PS and absence of Visceral Uptake being the primary indicators. In Panel B, for ATTRv patients, while both tracers effectively identify the disease, the predictive weight of specific findings varies: VISUAL_PS ≥ 2 is highly significant in both cohorts, though more pronounced in the HMDP group. Extracardiac findings, particularly MiscU, maintain significant diagnostic relevance for the variant phenotype regardless of the radiotracer utilized

In specific ATTR subtypes (Fig. 5), a VISUAL_PS ≥ 2 was confirmed as a highly significant predictor for the differential diagnosis of ATTRv versus AL in both the HMDP (*p* < 0.001) and DPD cohorts (*p* < 0.001). Similarly MiscU maintained a significant predictive value regardless of the tracer utilized, showing strong association in both DPD (OR 15.00, *p* < 0.001) and HMDP (OR 10.00, *p* = 0.017) populations. For ATTRwt diagnosis, predictive patterns were highly comparable between tracers: a VISUAL_PS ≥ 2 confirmed its robust diagnostic yield (DPD: OR 95.20, *p* < 0.001; HMDP: OR 30.75, *p* < 0.001), while Visceral Uptake maintained its role as a significant negative predictor for ATTRwt, consistently favoring an AL diagnosis across both the DPD (OR 0.11, *p* = 0.018) and HMDP (*p* = 0.001).

**Fig. 6:**
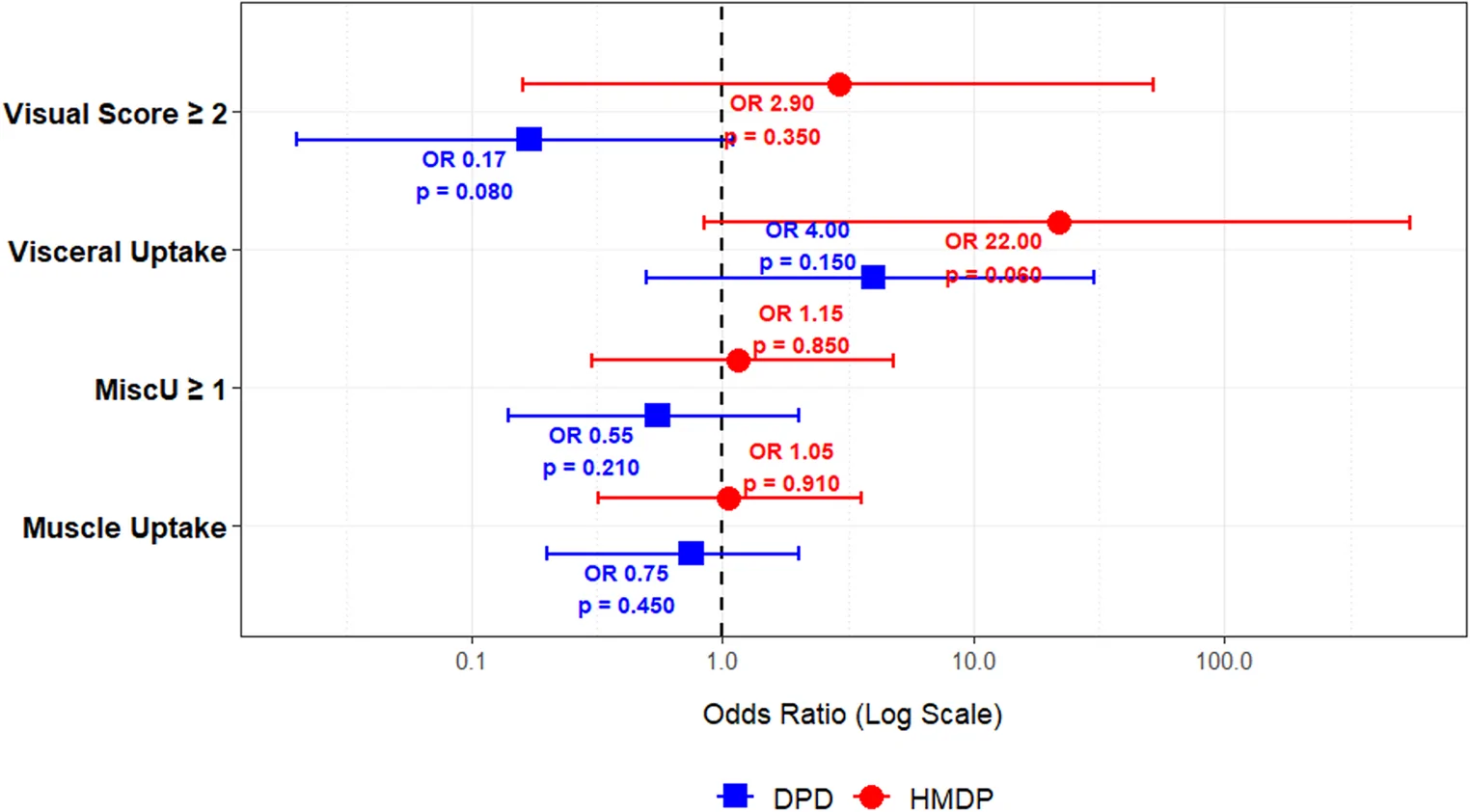
Odds Ratio Plot for the differential diagnosis model between ATTRv and ATTRwt subtypes. The DPD cohort (Blue) displays modest predictive values for distinguishing the two ATTR forms based on extracardiac distribution alone. In the HMDP cohort (Red), extreme values (such as those observed for High VISUAL_PS and Visceral Uptake) reflect a statistical separation phenomenon: since these features are nearly ubiquitous (VISUAL_PS) or consistently absent (Visceral Uptake) in both ATTR subtypes within the HMDP sample, they lack discriminatory power for differential staging, despite their high sensitivity for the general ATTR diagnosis

Finally, the multivariate logistic regression model for the direct differential diagnosis between ATTRv and ATTRwt subtypes demonstrated non-significant predictive values for all evaluated scintigraphic parameters across both radiotracer cohorts (Fig. 6). In the [99mTc]Tc-DPD cohort, the point estimates for the odds ratios (OR) clustered near the line of no effect, with wide 95% confidence intervals (CI) intersecting 1.0 for Visual Score ≥ 2 (OR 0.17, 95% CI: 0.02–1.10), Visceral Uptake (OR 4.00, 95% CI: 0.50–30.00), MiscU ≥ 1 (OR 0.55, 95% CI: 0.14–2.00), and Muscle Uptake (OR 0.75, 95% CI: 0.20–2.00). Similarly, within the [99mTc]Tc-HMDP cohort, all parameters crossed the reference line (OR = 1.0) and exhibited highly expanded confidence intervals, yielding an OR of approximately 2.90 (95% CI: 0.16–52.00) for Visual Score ≥ 2, 22.00 (95% CI: 0.85–550.00) for Visceral Uptake, 1.15 (95% CI: 0.30–4.80) for MiscU ≥ 1, and 1.05 (95% CI: 0.32–3.60) for MU.

## Discussion

**Fig. 7:**
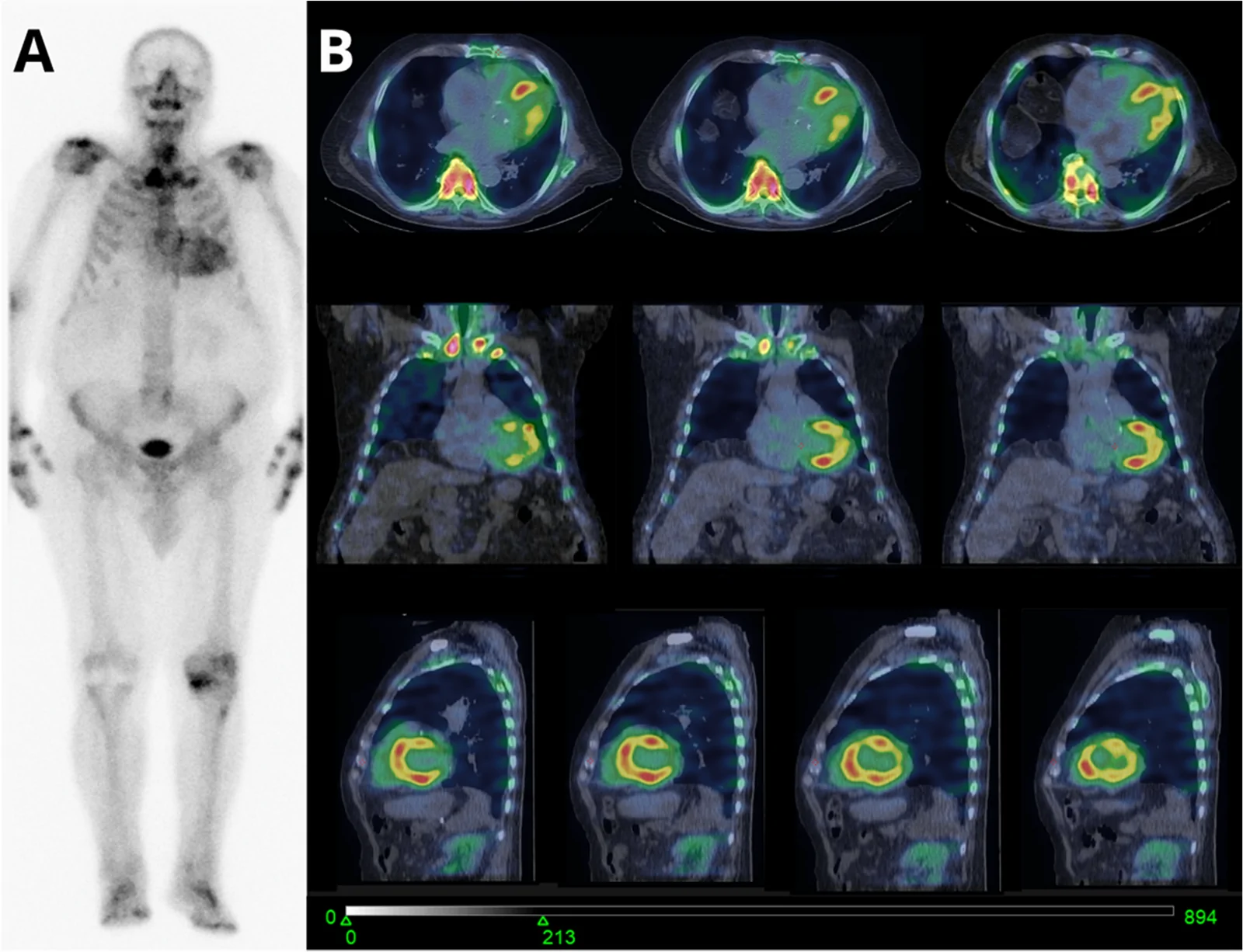
[99mTc]Tc-HMDP whole-body scintigraphic phenotype observed in the Florence cohort (ATTRwt subtype). (**A**) Whole-body planar scintigraphy showing intense biventricular cardiac uptake accompanied by soft-tissue fixation. (**B**) Corresponding cross-sectional SPECT/CT fusion images (transaxial, coronal, and sagittal planes) confirming intense myocardial tracer retention without evidence of pulmonary uptake

**Fig. 8:**
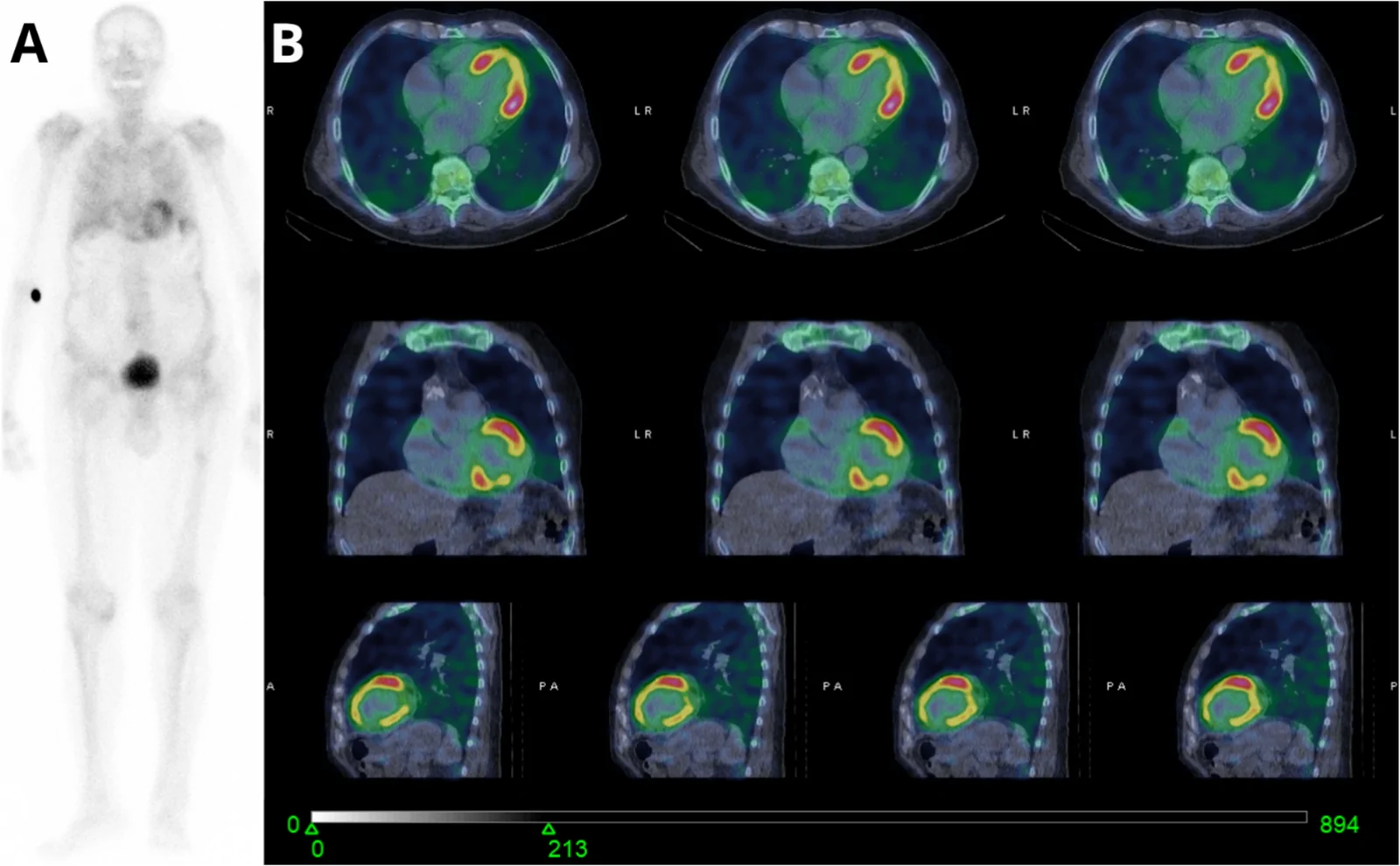
Multimodality assessment of tracer distribution in cardiac amyloidosis. (**A**) Whole-body planar scintigraphy showing intense biventricular myocardial uptake alongside a diffuse, subtle radiotracer retention throughout the lung fields. (**B**) Dedicated SPECT/CT cross-sectional fusion images (transaxial, coronal, and sagittal planes) isolating the myocardial signal to confirm its precise intracardiac localization while anatomically validating the diffuse pulmonary uptake observed on planar images, distinguishing it from technical artifacts or unrelated parenchymal lung disease

This multicenter study challenges the conventional assumption that bone-avid radiotracers function as fully interchangeable diagnostic agents in CA. Our data elucidate distinct biodistribution profiles between [99mTc]Tc-DPD and [99mTc]Tc-HMDP that directly impact visual phenotyping. While the established class-effect ensures high diagnostic accuracy for ATTR amyloidosis regardless of the compound used, the choice of radiotracer introduces specific pharmacokinetic variables, namely, the “soft-tissue sink” effect and selective pulmonary retention, that clinicians must carefully navigate to avoid diagnostic pitfalls. A central finding of our analysis is the systematic variance in myocardial grading between the two cohorts. We observed a marked skew towards Perugini Grade 3 within the HMDP group, whereas the DPD cohort was predominantly characterized by Grade 2 uptake. Crucially, this divergence must be interpreted alongside true negative cases (Perugini Grade 0), representing the absence of cardiac tracer avidity. The predominance of Grade 2 in DPD does not reflect a true disparity in overall disease severity or tracer sensitivity; rather, it arises from the distinctive whole-body kinetics of the two tracers. [99mTc]Tc-DPD exhibits a high affinity for amyloid deposits located in the appendicular skeletal musculature. This extensive soft-tissue retention generates a competitive “background” that attenuates the heart-to-body contrast, frequently keeping the visual score at Grade 2 even in advanced disease stages. Conversely, the rapid clearance of [99mTc]Tc-HMDP from soft tissues results in a higher target-to-background ratio, visually amplifying myocardial intensity and predisposing the scan to a Grade 3 classification (Fig. 7). Given these tracer-specific background variations, our findings highlight why SPECT/CT acquisitions are so important in daily practice. By providing precise anatomical localization and mitigating the superimposition of overlying soft-tissue activity, SPECT/CT is essential for resolving diagnostic ambiguities in myocardial grading, particularly when the competitive “sink effect” of DPD might otherwise lead to an underestimation of the true amyloid burden on planar imaging alone (Fig. 8). This kinetic divergence helps explain why our multivariate models did not directly differentiate between the ATTRv and ATTRwt subtypes based on scintigraphic parameters. The lack of statistical significance and the expanded confidence intervals observed in the direct comparison model do not imply biological identity between the two conditions; rather, they reflect the fact that both tracers operate at their upper and lower limits of pharmacokinetic sensitivity. Furthermore, epidemiological and referral biases inherent to retrospective cohorts must be considered: while ATTRwt patients frequently enter scintigraphic pathways via orthopedic evaluations (e.g., carpal tunnel syndrome or spinal stenosis), ATTRv cohorts often reflect a distinct referral pattern shaped by family screening programs and cascade testing. In the HMDP cohort, the near-ubiquity of a high VISUAL_PS paired with the total absence of visceral avidity in both subtypes mathematically prevents the model from calculating an independent staging weight, despite their high diagnostic sensitivity for the general ATTR phenotype. Looking beyond the myocardium, the study underscores that extracardiac uptake constitutes a reproducible biomarker of systemic disease rather than incidental background noise. Corroborating the foundational observations by Hutt et al., we observed that DPD retention in the gluteal and shoulder girdle musculature is a phenotypic hallmark of ATTR [19]. Consequently, reporting paradigms might need to evolve: when using DPD, skeletal muscle uptake should be actively assessed as a confirmatory sign of systemic amyloid burden, whereas its absence with HMDP does not preclude extracardiac involvement. Conversely, [99mTc]Tc-HMDP exhibited a distinct predilection for pulmonary retention. Aligning with data from Cappelli et al., we confirmed that lung uptake, quantified via the Janssen Score, is a prevalent feature of oxidronate imaging [20]. Recognizing this “pulmonary signature” is critical for the reporting physician to avoid misinterpreting lung retention as a technical artifact or an unrelated interstitial lung disease. Importantly, these pharmacokinetic nuances did not compromise the diagnostic performance in excluding AL amyloidosis. Neither tracer showed significant myocardial uptake in patients with light-chain disease, maintaining a robust negative predictive value. Furthermore, visceral uptake (hepatic and splenic) emerged as a tracer-independent indicator overwhelmingly favoring an AL diagnosis, reaffirming that while musculoskeletal and pulmonary retention are modulated by radiotracer chemistry, visceral avidity is driven by the intrinsic biology of fibril.

Crucially, while these scintigraphic signatures and extracardiac distribution profiles offer substantial diagnostic utility, they must not be interpreted as standalone predictors of the amyloid subtype. Our findings were derived from a retrospective cohort where diagnoses were rigorously established adhering to validated, non-invasive diagnostic algorithms. Consequently, a positive bone scintigraphy, even presenting with a Visual_PS ≥ 2 and characteristic extracardiac uptake, must invariably be contextualized within the broader clinical picture. Such scintigraphic patterns may occasionally present as incidental findings in bone scans performed for unrelated oncological or rheumatological indications, or in asymptomatic genetic carriers lacking overt cardiomyopathy. Therefore, these phenotypic markers should be conceptualized as probabilistic indicators that complement, rather than circumvent, the mandatory diagnostic workflow, which definitively requires the biochemical exclusion of monoclonal gammopathy and confirmatory genetic sequencing.

Finally, regarding study limitations, the retrospective nature of the analysis must be acknowledged as a potential source of selection bias. Furthermore, the use of different gamma camera models across centers (GE Discovery NM/CT 670 vs. 870) in a retrospective multicenter design, without a prior formal cross-validation protocol using phantoms, introduces a minor technical variable that could theoretically influence borderline semi-quantitative measurements, although core biological macro-trends remained robustly unaffected. In addition, the restriction to a specific subset of genetic mutations, primarily the Ile68Leu variant (which is the most prevalent in our study population), represents a constraint on the generalizability of results to other variants. However, this homogeneity simultaneously constitutes a methodological strength, as it minimizes the confounding variables often present in diverse genetic cohorts, allowing for a more rigorous evaluation of the tracers’ intrinsic behavior.

Addressing the translation of these findings to day-to-day clinical practice, our data indicate that reporting physicians must shift away from a generic, tracer-blind reading strategy. In routine clinical workflows, recognizing that DPD natively tends to stabilize scores at Grade 2 due to soft-tissue background kinetics prevents the misinterpretation of advanced disease as early-stage involvement, while identifying HMDP-associated pulmonary retention prevents confusion with secondary lung pathologies. Integrating these tracer-specific pharmacokinetic profiles into daily reporting protocols is essential for optimizing diagnostic accuracy and phenotyping.

In conclusion, while [99mTc]Tc-DPD and [99mTc]Tc-HMDP remain the gold standards for non-invasive diagnosis, they are distinct molecular entities. [99mTc]Tc-HMDP tends to maximize the “heart-to-bone” visual signal, potentially leading to higher visual grading, whereas [99mTc]Tc-DPD provides a more comprehensive, reliable view of the systemic muscular burden. Awareness of these nuances is essential for the expert reader to interpret scans accurately, particularly when subtle extracardiac findings may hold the key to the final diagnosis.

## Conclusions

This multicenter analysis confirms the high diagnostic accuracy of both [99mTc]Tc-DPD and [99mTc]Tc-HMDP for the non-invasive identification of transthyretin cardiac amyloidosis. However, our results conclusively demonstrate that these radiotracers possess distinct biodistribution profiles that preclude their direct interchangeability in visual grading and extracardiac assessment.

While [99mTc]Tc-HMDP provides a higher heart-to-background contrast, often favoring a Perugini Grade 3 classification, the significant soft-tissue retention of [99mTc]Tc-DPD tends to stabilize the visual score at Grade 2, reflecting a more prominent “sink effect” in the skeletal musculature. Furthermore, each tracer exhibits a unique systemic signature: [99mTc]Tc-DPD allows for a comprehensive and highly reproducible evaluation of the muscular amyloid burden, whereas [99mTc]Tc-HMDP is distinctively associated with pulmonary retention, a feature that must be recognized to avoid misinterpretation.

Importantly, both tracers maintained robust specificity in excluding AL amyloidosis, with hepatic and splenic uptake serving as critical tracer-independent red flags for light-chain disease.

In clinical practice, these findings suggest that the choice of radiotracer should influence the interpretive approach. Physicians must move beyond a generic reading strategy, adopting a tracer-specific perspective that accounts for these intrinsic pharmacokinetic differences to ensure accurate diagnosis and phenotyping.

## Data Availability

The datasets generated during and/or analyzed during the current study are available from the corresponding author for reasonable requests. This study was performed in line with the principles of the Declaration of Helsinki. This is an observational study. The Ethics Committee of IRCCS Azienda Ospedaliero-Unversitaria Policlinico S. Orsola di Bologna Research has confirmed that no ethical approval is required.
